# Repertoire and Diversity of Toxin – Antitoxin Systems of Crohn’s Disease-Associated Adherent-Invasive *Escherichia coli*. New Insight of T his Emergent *E. coli* Pathotype

**DOI:** 10.3389/fmicb.2020.00807

**Published:** 2020-05-06

**Authors:** Paula Bustamante, Roberto Vidal

**Affiliations:** ^1^Programa de Microbiología y Micología, Instituto de Ciencias Biomédicas (ICBM), Facultad de Medicina, Universidad de Chile, Santiago, Chile; ^2^Instituto Milenio de Inmunología e Inmunoterapia, Facultad de Medicina, Universidad de Chile, Santiago, Chile

**Keywords:** toxin–antitoxin systems, AIEC, adherent invasive *E. coli*, mobile genomic elements, pathogenicity island, enteric pathogenic bacteria

## Abstract

Adherent-invasive *Escherichia coli* (AIEC) corresponds to an *E. coli* pathovar proposed as a possible agent trigger associated to Crohn’s disease. It is characterized for its capacity to adhere and to invade epithelial cells, and to survive and replicate inside macrophages. Mechanisms that allow intestinal epithelium colonization, and host factors that favor AIEC persistence have been partly elucidated. However, bacterial factors involved in AIEC persistence are currently unknown. Toxin–antitoxin (TA) systems are recognized elements involved in bacterial persistence, in addition to have a role in stabilization of mobile genetic elements and stress response. The aim of this study was to elucidate the repertoire and diversity of TA systems in the reference AIEC NRG857c strain and to compare it with AIEC strains whose genomes are available at databases. In addition, toxin expression levels under *in vitro* stress conditions found by AIEC through the intestine and within the macrophage were measured. Our results revealed that NRG857c encodes at least 33 putative TA systems belonging to types I, II, IV, and V, distributed around all the chromosome, and some in close proximity to genomic islands. A TA toxin repertoire marker of the pathotype was not found and the repertoire of 33 TA toxin genes described here was exclusive of the reference strains, NRG857c and LF82. Most toxin genes were upregulated in the presence of bile salts and acidic pH, as well as within the macrophage. However, different transcriptional responses were detected between reference strains (NRG857c and HM605), recalling the high diversity associated to this pathotype. To our knowledge this is the first analysis of TA systems associated to AIEC and it has revealed new insight associated to this emergent *E. coli* pathotype.

## Introduction

Infectious agents are thought to influence and to be determinants of chronic diseases (O’Connor et al., 2006). In particular, Crohn’s disease (CD), a chronic inflammatory disease of the gastrointestinal tract, is thought to result from interactions between resident microbes and the host innate immune system, in genetically susceptible individuals; however, its exact etiology is still unclear (Torres et al., 2017). Two decade ago the group headed by Arlette Darfeuille-Michaud discovered a new pathotype of *Escherichia coli* associated with CD termed adherent-invasive *E. coli* (AIEC) (Darfeuille-Michaud et al., 1998; Boudeau et al., 1999). Nowadays, AIEC is the most likely candidate to cause specific damage to people who are genetically susceptible to the development of CD, and in consequence it has been proposed as a possible agent trigger for this disease (Mirsepasi-Lauridsen et al., 2019). AIEC does not encode classic virulence genes associated to other diarrheagenic *E. coli* pathotypes, instead genomic studies have confirmed that AIEC resemble extraintestinal pathogenic *E. coli* (ExPEC) as they share -to a larger extent- similar virulence gene sets and certain strains are phylogenetically related (Martinez-Medina et al., 2009). However, AIEC possesses unknown virulence-specific features as these strains are characterized by their capacities to adhere and invade epithelial cells, and to survive and replicate within macrophages without triggering host cell death (Glasser et al., 2001).

Two strains, LF82 and NRG857c, have been classically considered the reference AIEC strains; they belong to the same phylogroup (B2) and serotype (O83:H1), and are genetically very close as their genomes are highly similar, despite to carry different plasmids and antibiotic resistance genes (Miquel et al., 2010; Nash et al., 2010). Genomic islands have also been described on the NRG857c chromosome, in many cases highly orthologous in LF82 but weakly conserved or absent in other *E. coli* pathotypes and commensal organisms (Nash et al., 2010), suggestive that these genomic islands may have an influential role in the expression of the AIEC phenotype; however, their contribution to pathogenicity has not been explored. The HM605 strain (B2, O1:H7) has also been considered as a reference AIEC strain for some authors, but this strain is phylogenetically distant of NRG857c and LF82 (Clarke et al., 2011). Actually, a growing body of work has revealed that AIEC strains are phylogenetically heterogeneous (Cespedes et al., 2017; O’Brien et al., 2017; Rakitina et al., 2017), and no gene or sequence exclusive to the AIEC pathotype has yet been identified.

Pathogenicity mechanisms of AIEC have mainly been studied in the LF82 strain and several important factors have been identified (Yang et al., 2017), such as type 1 pili (FimH) and flagella which facilitate binding to and invasion of the epithelial cell; long polar fimbriae that aids in the binding to M cells overlying Peyer’s patches; Vat protease that promotes mucins degradation, as well as the stress protein HtrA (Bringer et al., 2005) and the oxidoreductase DsbA (Bringer et al., 2007), which are required for replication within macrophages. The expression of most of those virulence factors is modulated by bile salts (Chassaing et al., 2013; Delmas et al., 2019) and the acidic conditions found inside phagolysosomes (Bringer et al., 2005, 2007), suggesting that these stresses may be environmental signals to promote AIEC colonization. However, genes related to its pathogenicity are not AIEC-specific and are present in the majority of *E. coli* strains, including non-pathogenic strains. In addition, bacterial factors involved in AIEC persistence are currently unknown, though recently it was revealed that LF82 relies on SOS and stringent responses to survive, replicate and tolerate antibiotics within macrophages (Demarre et al., 2019). Therefore, the identification of additional genetic elements or the differential expression of key genes that could be involved in AIEC pathogenicity represent important milestones that must be achieved.

Toxin–Antitoxin (TA) systems have emerged as potential virulence factors, not only affecting pathogenicity, but also involved in biofilm formation and persistence (Wang and Wood, 2011; Kedzierska and Hayes, 2016; Lobato-Marquez et al., 2016a), in addition to have a role in stabilization of mobile genetic elements (MGE) and stress response (Gerdes et al., 2005). TA systems correspond to bacterial small genetic elements located on plasmids and chromosomes, diverse in sequence, antitoxin nature and mechanistic, and currently classified into seven types depending on the nature and activity of the antitoxins (all known TA toxins are proteins) (Marimon et al., 2016; Harms et al., 2018; Song and Wood, 2018). In types II, IV, V, VI, and VII the antitoxin is a protein that forms a stable non-toxic protein complex with the toxin, acts as a toxin antagonist, cleaves toxin mRNA, facilitates toxin degradation or inactivates the toxin by oxidizing a cysteine residue, respectively. However, in types I and III TA systems, antitoxins correspond to non-coding RNAs that interact with the toxin transcripts or with the toxic protein, respectively (Brantl and Jahn, 2015). Most bacterial chromosomes contain multiple TA systems that are commonly considered part of the accessory genome, as some of them are prone to horizontal gene transfer and intragenomic recombination (Ramisetty and Santhosh, 2016), as well as to regulatory crosstalks (Wang et al., 2013; Wessner et al., 2015).

Known stresses found by pathogens inside their hosts, such as nutrient starvation, acidic pH and oxidative stress, are common to those some TA systems are responsive (Lobato-Marquez et al., 2016a). TA systems provide niche-specific benefits within the host and enhances the stress resistance of uropathogenic *E. coli* (UPEC) (Norton and Mulvey, 2012). *Haemophilus influenzae* uses type II TA systems to colonize and survive to stress in animal organs (Ren et al., 2012) and their TA loci are induced after infection of mucosal intestinal epithelia (Baddal et al., 2015). Similarly, *Vibrio cholerae* induces type II TA systems in virulence-inducing conditions, and some of them are required for efficient host colonization (Wang et al., 2015). *Mycobacterium tuberculosis* (Korch et al., 2009) and *Salmonella* (Helaine et al., 2014) up-regulate genes encoding TA systems inside macrophages, while distinct type I and type II TA systems control *Salmonella* lifestyle inside eukaryotic cells (Lobato-Marquez et al., 2015). In addition, TA systems are involved in *Salmonella* persister formation during macrophage infection (Helaine et al., 2014). On the other hand, as pathogenic bacteria could carry plasmids and pathogenicity islands (or other MGE) encoding virulence factors, TA systems could indirectly participate in virulence having the potential to contribute to the maintenance of these genetic elements in the genome (Sayeed et al., 2005; Garcia-Quintanilla et al., 2006; Lobato-Marquez et al., 2016b).

In particular, TA systems have been linked to responses to bile acids (Kwan et al., 2015) and acid pH (Helaine et al., 2014), same types of stresses faced by AIEC into the host and known to induce their virulence genes. However, to our knowledge, no TA systems analyses have been done in the AIEC pathotype.

Here we describe the content and diversity of TA systems in AIEC and the effect of gastrointestinal and intramacrophage growth conditions on their TA toxins expression. The analysis of the NRG857c genome revealed a high number of putative TA systems distributed through the entire chromosome and some of them associated to genomic islands. A comparative analysis showed that most TA systems are common between AIEC and non-AIEC strains and they cluster essentially according to phylogroup. *mazF-1* toxin was the only one found associated exclusively to some AIEC strains but not as a marker of the pathotype. Toxin genes were transcriptionally active and upregulated in the presence of bile salts and acid pH, as well as within the macrophage. To our knowledge this is the first analysis of TA systems encoded by AIEC and would help to a better comprehension of the pathogenicity mechanisms of this *E. coli* pathotype.

## Materials and Methods

### *In silico* Identification of Putative TA Systems

As all toxin genes from TA systems are known to encode proteins, we focused our search on them. For those identified TA systems that have RNA as antitoxins, we did not go deep in their characterization, as it was not the focus of this work. However, proteinaceous antitoxins were included in the *in silico* analysis.

Toxin nucleotide sequences from all TA types were downloaded from the database TADB version 2.0 (Xie et al., 2018) on June 2017; only sequences from experimentally validated systems were retrieved. CD-Hit suite (Huang et al., 2010) was used to eliminate redundancy from our TA database. NRG857c chromosome (GenBank NC_017634) was inspected for TA toxins genes using the Large Scale Blast Score Ratio (LS-BSR) pipeline (Sahl et al., 2014) and our *in house* TA database. Alignments were carried on by TBLASTN using default parameters, but (as per the small size of TA proteins) short peptides (<50 amino acids) were not filter out during the process, and only peptides of a length smaller than 15 amino acids (instead of the default value of 33 amino acids) were discarded after translating sequences. LS-BSR score ratios range from 0 to 1 where values above 0.7 were considered strong positive hits. With the LS-BSR output the NRG857c genome was manually inspected by TBLASTN or BLASTN to identify each TA locus and to define the TA repertoire. The type II toxins result was also contrasted with the output of type II TA systems retrieved by the TAfinder web server^1^.

*dinQ*/*agrB*, *tisB*/*istR*, *ibs*/*sib*, and *ralRA* type I TA systems and the type VI SocAB system were not included in the TADB database, so these genes were search individually by BLASTN.

### General Bioinformatic Tools

Resources available at NCBI (i.e., BLAST, ORFfinder, and CD-Search) were used for general bioinformatic analyses. PCR primers were designed using the NCBI software toll Primer-BLAST (Ye et al., 2012). Multiple sequence alignments were carried on by Clustal Omega (Sievers et al., 2011). ISfinder (Siguier et al., 2006) was used to search for bacterial insertion sequences. The LS-BSR pipeline (Sahl et al., 2014) was used to screen TA toxins between bacterial genomes as described above. The genome sequences used at the comparative analyses are described at Supplementary Table S1. Genome comparisons were done and visualized using EasyFig 2.2.2 (Sullivan et al., 2011). Heatmaps and plots were drawn in Prism 8 and edited with Adobe Photoshop 8 software. The genomic map of TA systems in the NRG857c chromosome was created with the SnapGene Viewer software (from GSL Biotech^2^). Principal Component Analysis (PCA) plots and heatmaps were done using the web tool ClustVis (Metsalu and Vilo, 2015).

### Bacterial Strains and Growth Conditions

The AIEC reference strains NRG857c and HM605 were grown routinely in Luria-Bertani Lennox (LB) medium (BD Difco) at 37°C with agitation. When needed, the medium was supplemented with 2% bile salts (BD Difco Bile Salt N. 3) or adjusted to pH 4.5 with HCl.

### Treatment of AIEC With Bile Salts and Acid pH

Fresh LB medium was inoculated 1/100 from an overnight culture and incubated with agitation at 37°C per 2 h until early exponential phase. Pre-warmed fresh LB medium containing 2% bile salts or LB adjusted at pH 4.5 was inoculated 1/20 using the exponential phase culture as inoculum. Cultures were incubated for additional 2 h at 37°C with agitation and aliquots were taken for RNA extraction as described below. All culture treatments were done in triplicate.

### Macrophage Cell Culture Infection

The J774.A1 murine macrophage cell line (murine peritoneal macrophages; ATCC TIB-67) was maintained in high glucose Dulbecco’s Modified Eagle (DMEM) medium (Hyclone) supplemented with 10% heat-inactivated fetal bovine serum (PanBio) and penicillin/streptomycin (Gibco), and grown at 37°C in a humidified atmosphere with 5% CO_2_ with regular media changes. For infection assays, J774.A1 macrophages were seeded at 9.5 × 10^5^ cells per well of a 6-well plate (Nunc) 24 h prior to infection. Infections were carried out in high glucose DMEM medium at a multiplicity of infection of 10 bacteria per macrophage, in duplicates. After 10 min of centrifugation at 900 × *g* and a 10 min incubation period at 37°C with 5% CO_2_, the infected macrophages were washed twice with phosphate buffer saline (PBS; Merck), and fresh cell culture medium containing 150 μg/ml of amikacin (Sigma) was added to kill extracellular bacteria (time 0 of infection). After 1 h of incubation at 37°C with 5% CO_2_, the medium was removed, infected cells washed twice with PBS and total RNA was extracted as described below.

### Total RNA Extractions

RNA was extracted using the phenol-based reagent TRIsure (Bioline). For bacterial culture, cells were centrifuged at 7,500 × *g* 10 min at 4°C, pellets resuspended in 1 ml of TRIsure and the manufacturer’s instruction were followed. For infected macrophages, 500 μl of TRIsure were added to each well and incubated at 4°C per 2 h to lyse and detach the cells. The TRIsure lysed of two wells (corresponding to the same sample) was combined and the manufacture’s protocol was followed.

RNA pellets were resuspended with DEPC-treated water (Sigma) and stored at −80°C. RNA concentration and integrity were determined by UV spectrophotometry and agarose gel electrophoresis, respectively.

### Quantitative Reverse Transcription PCR (qRT-PCR)

cDNAs were synthesized using 1.5 μg of DNase I-treated RNA (DNA-free kit, Ambion), random primers and the High-Capacity cDNA Reverse Transcription kit (Applied Biosystems), according to the manufacturer’s protocol. A 1/10 dilution of each cDNA sample was used as template for real time PCR (qPCR) and primer pairs listed at Supplementary Table S2. Reactions were prepared with the Brilliant II SYBR Green qPCR Master Mix (Agilent Technologies) and carried on using a fast protocol with two steps cycling (5 min at 95°C; 40 cycles of 10 s at 95°C and 30 s at 60°C; followed by a melting curve between 65 and 95°C with 5 s per step of 0.5°C increments) in the AriaMx Real-Time PCR System (Agilent Technologies). Relative expressions were expressed as fold changes against the level of expression in LB medium at early exponential phase and determined using the efficiency-calibrated ΔCt model (Pfaffl, 2001) using *gapA* as a reference gene. Primer amplification efficiencies were determined from the slope of standard curves generated with five points of NRG857c genomic DNA serial dilutions.

## Results

### The Reference AIEC NRG857c Strain Carries a High Number of Putative TA Systems

**FIGURE 1:**
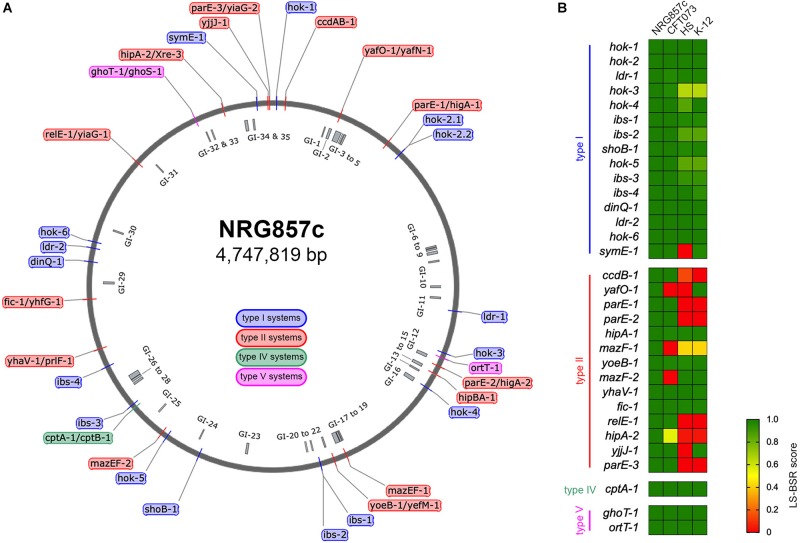
Repertoire of putative TA systems in AIEC NRG857c. **(A)** Chromosomal position of the 33 putative TA systems identified in the NRG857c chromosome. TAs are indicated with a color code according to the legend shown inside the main circle. The position of 35 genomic islands (GI) is shown inside the main circle in gray. **(B)** Screening of the 33 putative TA systems from NRG857c in *Escherichia coli* UPEC CFT073, HS and K-12 (MG1655). As per *hok-2.1* and *hok-2.2* are identical, they were analyzed as a single gene (*hok-2*). Genome sequences of CFT073 (Genbank NC_004431), HS (Genbank NC_009800), and K-12 MG1655 (Genbank NC_000913) were examined for its presence or absence of NRG857c toxins genes by TBLASTN using the LS-BSR pipeline. Achieved LS-BSR score ratios are plotted as a heatmap according to the scale shown at the legend.

Using our *in house* TA database, the NRG857c chromosome was inspected for its content of TA genes. After manual curation, a total of 33 putative TA systems were identified (Table 1 and Figure 1), accounting for 16 type I, 14 type II, 1 and 2 representatives of types IV and V TA systems, respectively.

**TABLE 1:** Putative toxin–antitoxin systems encoded by the AIEC reference strain NRG857c (for type I TA systems only the toxin characteristics are shown).

**Type**	**TA no.**	**Locus**	**Location^#^**	**Conserved Domain (Accession)^&^**	**Given name**	**Comments**
I	TA1	NRG857_00085	c15,471.15,623	HOK_GEF Superfamily (cl27487, pfam01848)	Hok-1	Homologous to *hokC* in *E. coli* K12; close to GI
I	TA2	NRG857_02625′	577,301.577,453^$^	HOK_GEF Superfamily (cl27487, pfam01848)	Hok-2.1	Homologous to *hokE* in *E. coli* K12
I	TA3	NRG857_02630	577,805.577,957	HOK_GEF Superfamily (cl27487, pfam01848)	Hok-2.2	Duplication of *hok-2.1*
I	TA4	NRG857_06225′	c1,288,750.1,288,857^$^	Ldr_toxin Superfamily (cl16489, pfam13940)	Ldr-1	Homologous to *ldr-A*, *-B* and *-C* from *E. coli* K-12
I	TA5	Not annotated	c1,466,912.1,467,052	HOK_GEF Superfamily (cl27487, PRK09738)	Hok-3	Homologous to *hokB* in K-12
I	TA6	NRG857_07840′	c1,626,517.1,626,672^$^	HOK_GEF Superfamily (cl27487, pfam01848)	Hok-4	Homologous to *hokD* in K-12
I	TA7	Not annotated	c2,175,707.2,175,763	NI	Ibs-1	Homologous to *ibsA* in K-12
I	TA8	Not annotated	c2,176,038.2,176,091	NI	Ibs-2	Homologous to *ibsB* in K-12
I	TA9	Not annotated	c2,685,649.2,685,729	NI	ShoB-1	
I	TA10	NRG857_13500	c2,838,049.2,838,201	HOK_GEF Superfamily (cl27487, pfam01848)	Hok-5	Homologous to *hokX* in *E. coli* B
I	TA11	Not annotated	c3,031,097.3,031,156	NI	Ibs-3	Homologous to *ibsC* in K-12
I	TA12	Not annotated	3,220,142.3,220,201	NI	Ibs-4	Homologous to *ibsE* in K-12
I	TA13	Not annotated	c3,663,342.3,663,425	NI	DinQ-1	Homologous to dinQ in K-12
I	TA14	NRG857_17585	c3,720,245.3,720,352	Ldr_toxin Superfamily (cl16489, pfam13940)	Ldr-2	Homologous to *ldr-D* in K-12
I	TA15	NRG857_17710	c3,746,235.3,746,387	HOK_GEF Superfamily (cl27487)	Hok-6	Homologous to *hokA* in K-12
I	TA16	NRG857_21965	c4,681,978.4,682,319	SymE_toxin Superfamily (cl07446, PRK13605)	SymE-1	Close to RM cluster genes
II	TA17	NRG857_00260	52,569.52,883	PemK_toxin Superfamily (cl00995, pfam01845)	CcdB-1	Genomic islet
		NRG857_00255	52,333.52,566	CcdA Superfamily (cl02188, COG5302)	CcdA-1	
II	TA18	NRG857_01270	283,709.284,107	YafO_toxin Superfamily (cl08066, PRK09885)	YafO-1	
		NRG857_01265	283,413.283,706	PhdYeFM_antitox Superfamily (cl09153, PRK09778)	YafN-1	
II	TA19	NRG857_02300	508,648.508,833	ParE_toxin Superfamily, HigB (cl21503, COG3549, pfam05015)	ParE-1	
		NRG857_02305	508,869.509,210	HTH_XRE Superfamily, antidote_HigA (cl22854, TIGR02607)	HigA-1	
II	TA20	NRG857_07325	c1,526,513.1,526,791	ParE_toxin Superfamily (cl21503)	ParE-2	
		NRG857_07320	c1,526,229.1,526,513	HTH_XRE Superfamily, antidote_HigA (cl22854, TIGR02607)	HigA-2	
II	TA21	NRG857_07490	c1,561,146.1,562,468	HipA_C Superfamily (cl26849, COG3550)	HipA-1	
		NRG857_07495	c1,562,468.1,562,734	HTH_XRE Superfamily, antitoxin HipB (cl22854, PRK09726)	HipB-1	
II	TA22	NRG857_10010	c2,063,636.2,063,968	PemK_toxin Superfamily (cl00995)	MazF-1	Exclusive of AIEC strains; encoded in a MGE integrated at the *asnV* gene
		NRG857_10015	c2,063,969.2,064,226	MazE_antitoxin Superfamily (cl00877)	MazE-1	
II	TA23	NRG857_10285	c2,117,235.2,117,489	ParE_toxin Superfamily, YoeB_toxin (cl21503, pfam06769)	YoeB-1	
		NRG857_10290	c2,117,486.2,117,737	PhdYeFM_antitox Superfamily, antitoxin YefM (cl09153, PRK11409)	YefM-1	
II	TA24	NRG857_13620	c2,863,299.2,863,634	PemK_toxin Superfamily, toxin MazF (cl00995, PRK09907)	MazF-2	Classic MazEF close to *relA*
		NRG857_13625	c2,863,634.2,863,882	MazE_antitoxin Superfamily, antitoxin MazE (cl00877, PRK09798)	MazE-2	
II	TA25	NRG857_15545	3,299,073.3,299,537	Toxin_YhaV Superfamily (cl20161, pfam11663)	YhaV-1	
		NRG857_15540	3,298,738.3,299,073	MazE_antitoxin Superfamily, putative regulator PrlF (cl00877, PRK09974)	PrlF-1	
II	TA26	NRG857_16665	c3,508,219.3,508,821	FIDO Superfamily, cell filamentation protein Fic (cl26489, PRK10347)	Fic-1	
		NRG857_16670	c3,508,811.3,508,978	DUF2559 Superfamily (cl23922, PRK10204)	YhfG-1	
II	TA27	NRG857_19405	4,121,686.4,121,997	NI	RelE-1	
		NRG857_19410	4,121,998.4,122,288	YiaG Superfamily (cl27792)	YiaG-1	
II	TA28	NRG857_21375	4,541,917.4,543,185	HipA_C Superfamily, HipA_C (cl26849, pfam07804) HipA_N (cl06714, pfam07805)	HipA-2	Exclusive of NRG857c; encoded close to a fatty acids metabolism cluster gene
		NRG857_21370	4,541,621.4,541,914	NI	Xre-3	
II	TA29	NRG857_22175	4,727,542.4,728,873	HipA_C suprfamily (cl26849, PRK09775)	YjjJ-1	Paralogous of *hipA*; the gene encoding YjjJ is present as a single gene and not in an operon.
II	TA30	NRG857_22205	4,734,411.4,734,743	ParE_toxin Superfamily (cl21503)	ParE-3	Genomic islet
		NRG857_22210	4,734,745.4,735,029	YiaG Superfamily (cl27792)	YiaG-2	
IV	TA31	NRG857_14215	c3,015,644.3,016,051	Cpta_toxin (cl06333, pfam07254)	CptA-1	
		NRG857_14220	c3,016,032.3,016,298	Sdh5 Superfamily (cl01110, PRK10878)	CptB-1	
V	TA32	NRG857_20710	4,420,135.4,420,308	Toxin_GhoT_OrtT (cl11347, pfam10753)	GhoT-1	
		NRG857_20705	4,419,811.4,420,107	GhoS Superfamily (cl12641)	GhoS-1	
V	TA33	NRG857_07135	1,488,479.1,488,727	Toxin_GhoT_OrtT (cl11347, pfam10753)	OrtT-1	Orphan type V toxin

**^#^A c letter preceding the nucleotide coordinates denotes that the gene is encoded by the reverse strand. ^&^According to Conserved Domain database (CDD) NCBI; NI: no conserved domain identified. ^$^Reannotated*.*

The putative TA systems were found distributed around the entire NRG857c chromosome, and some in close proximity to the genomic islands described by Nash et al. (2010) (Figure 1A, GI-1 to GI-35). We noticed some genes that had escaped the annotation or had been incorrectly annotated and they are described at Supplementary Material. None of the genes identified, except *hok-2*, encode identical TAs. Original names were assigned to those TA systems displaying high sequence homology to those previously characterized or carrying functional domains of known toxins/antitoxins.

As AIEC resemble ExPEC in several characteristics, the TA content of NRG857c was initially compared with the reference UPEC CFT073 strain, as well as non-pathogenic *E. coli* strains (HS and K-12). The conservation of the NRG857c TA toxins repertoire between the genomes was explored by TBLASTN using the LS-BSR pipeline. Our analysis revealed that the occurrence of type II toxins shows the major variability (Figure 1B), in agreement with its recognized horizontal transfer heritage (Ramisetty and Santhosh, 2016). Seven type II toxin genes (*ccdB-1*, *parE-1*, *parE-2*, *mazF-1*, *relE-1*, *hipA-2*, and *parE-3*) are absent in both non-pathogenic *E. coli* strains, and four (*yafO-1*, *mazF-1*, *mazF-2*, and *hipA-2*) are not encoded by UPEC CFT073; hence, *mazF-1* and *hipA-2* toxin genes seem to be unique to NRG857c according to this preliminary screening (Figure 1B).

It is known that most bacteria encode multiple TAs, however, the diversity and accumulation of TAs on bacterial genomes and its physiological implications are highly debated (Goormaghtigh et al., 2018; Song and Wood, 2018). Our results indicate that the reference AIEC NRG857c strain carries a high number of putative TA systems, although lower than the TA repertoire reported for *E. coli* K-12 MG1655 (Harms et al., 2018).

### Types I, IV, and V Putative TA Toxins of NRG857c Are Well Conserved

Seven out 16 type I putative TA toxins on the NRG857c chromosome correspond to *hok* homologous. Only one toxin gene seems to be duplicated, named *hok-2.1* and *hok-2.2*, and NRG857c Hok proteins share between 21.7 and 82% amino acid identity (excluding the identical Hok-2). *E. coli* K-12 codes for five *hok* loci (denoted *hokA* to *hokE*) that are non-functional, either by inactivation by insertion elements, point mutations or genetic rearrangement (Pedersen and Gerdes, 1999). NRG857c codes all these five *hok* homologs and an additional *hok* gene (*hok-5*) at the intergenic *cysH*-*iap* region, which is homolog to one previously identified in *E. coli* B (*hokX*), also inactivated by an insertion element. DNA sequence analysis by ISfinder (Siguier et al., 2006) revealed that the corresponding NRG857c homologous, *hok-1* (*hokC*), *hok-2* (*hokE*), *hok-3* (*hokB*), *hok-4* (*hokD*), *hok-5* (*hokX*), and *hok-6* (*hokA*), seem to be intact loci lacking of IS elements (Supplementary Figure S1).

In addition to *hok* toxins, NRG857c encodes nine type I toxins belonging to Ldr, Ibs, ShoB, DinQ and SymE families. There are four Ldr sequences in *E. coli* K-12 (A, B, C, and D), three of them in tandem (LdrA, B and C) (Kawano et al., 2002). Ldr-1 is encoded between *chaA* and *kdsA* genes, as the *ldr-A*, *-B*, *-C* at *E. coli* K-12, but two of these systems are missed in NRG857c (Supplementary Figure S2A). Ldr-2 is encoded between *bcsG* and *yhjV* genes, same as its counterpart *ldr-D* in *E. coli* K-12 (Supplementary Figure S2B). Ldr-1 is 97.1% identical to Ldr-A, -B, and-C and Ldr-2 97.1% identical to Ldr-D. As *ldr-A*, *-B*, and *-C* share high identity and they are encoded in tandem, we speculate that the absent of two systems between *chaA* and *kdsA* genes in NRG857c could be due to a sequencing/assembly error.

SymE-1 is 94.7% identical to its counterpart at *E. coli* K-12, but seems to be encoded in a different context shared by the UPEC CFT073, however, in all of them the *symE* gene is close to restriction-modification (RM) operons that seem part of MGEs (Supplementary Figure S3). Indeed, it is well known that *symE* genes tend to associate with mobile elements such as transposons, RM modules and pathogenicity islands (Kawano et al., 2007).

We notice that some of the type I chromosomal TA toxins described for *E. coli* K-12 (i.e., *dinQ*/*agrB*, *tisB*/*istR*, *ibs*/*sib*, and *ralRA*) were missing from our TA database, so we searched them by BLASTN (Supplementary Material). The *dinQ* locus in *E. coli* K-12 is conserved in NRG857c, but the corresponding gene was missed from the annotation. Similarly, *E. coli* K-12 encodes five copies of the *ibs*/*sib* system (Fozo et al., 2008), but they were also missed from the NRG857c genome annotation. However, we were unable to find homologs to the *tisB*/*istR* and *ralRA* systems. A segment of approximately 600 nucleotides, that includes *tisB* at K-12, is missed from the NRG857c chromosome (not shown). Similarly, the *ralR* type I toxin in K-12 is encoded within an approximately 23 kbp chromosomal segment, that is absent in NRG857c chromosome, so is the *ralR* gene (not shown).

Thus, our analysis revealed that the putative type I TA toxins in AIEC NRG857c are syntenic with their counterparts in K-12, although there are some differences concerning to genomic contexts and some of them have been missed from the NRG857c annotation.

The screening of type I toxin genes on CFT073, HS and K-12 genomes, revealed that, with the exception of *symE-1* and *hok-3*, type I toxin genes are well conserved between the four tested strains (Figure 1B). However, due to some type I TA systems are present in more than one copy and they share high nucleotide similarity, and also considering the limitations of our screening strategy, we cannot conclusively discard differences between strains.

The type IV (CptAB-1 and TA32) and the type V (GhoST-1 and OrtT-1, TA33 and TA34, respectively) TA systems are well conserved and encoded at the same locus as in K-12, HS, and CFT073 strains.

### Common Type II TA Systems in NRG857c

**FIGURE 2:**
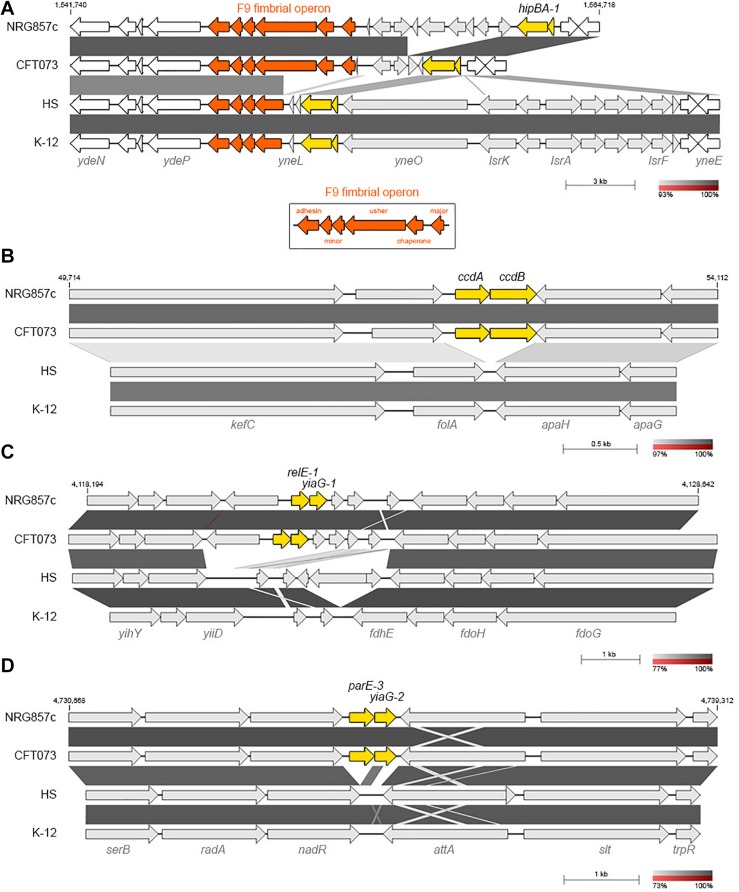
NRG857c encodes common and pathogen-associated putative TA systems. **(A)** Genomic comparison of chromosomal regions encoding for *hipBA-1*. F9 fimbrial operon is in orange; the intact F9 operon (shown in the bottom box) consists of six structural genes, encoding: the major subunit, chaperone, usher, two minor subunits and an adhesin. **(B–D)** Pathogen-associated type II TA systems. **(B)** Comparison of the *kefC*-*apaG* region covering the *ccdAB-1* system. **(C)** Comparison of the *relE-1*/*yiaG-1* locus. **(D)** Comparison of the *serB*-*trpR* region covering the *parE-3*/*yiaG-2* system. Genomes (same as Figure 1) were compared by BLASTN and highly homologous regions are shaded in gray colors according to the percentage of identity indicated at the legend shown below each figure. In each figure TA systems are highlighted in yellow and the coordinates of the DNA segment of NRG857c chromosome used for the comparison are indicated.

Only 4 out 14 type II TA toxin genes identified in NRG857c are shared by the four tested strains (*hipA-1*, *yoeB-1*, *yhaV-1*, and *fic-1*; Figure 1B). However, as it is known that the genetic context of individual TAs could vary, affecting their activities and/or roles (Ramisetty and Santhosh, 2016), we looked in detail the synteny of each individual TA locus. *yoeB-1*/*yefM-1* (TA23), *yhaV-1*/*prlF-1* (TA25), and *fic-1*/*yhfG-1* (TA26) are well conserved in their genetic context in all the analyzed strains (not shown). However, in spite of to be a common TA system, the genetic context of *hipBA-1* (TA21) is variable. *hipBA-1* is encoded upstream of a fimbrial operon (F9 operon, Figure 2A), encoding a chaperone-usher fimbriae (Wurpel et al., 2013). The immediate 5′ region of F9 operon is variable and contains a range of different insertions and/or deletions; noteworthy the *hipBA* locus is conserved (Figure 2A). A complete F9 operon seems to be present in pathogenic strains, NRG857c and CFT073; however, it is known that these loci are disrupted in many strains, as we noticed for HS and K-12. Remarkable, the intact F9 operon is highly prevalent in intestinal pathogenic *E. coli*, including AIEC (Wurpel et al., 2013), reflecting that it could has an important role for pathogenesis.

### Pathogen-Associated Type II TA Systems

**FIGURE 3:**
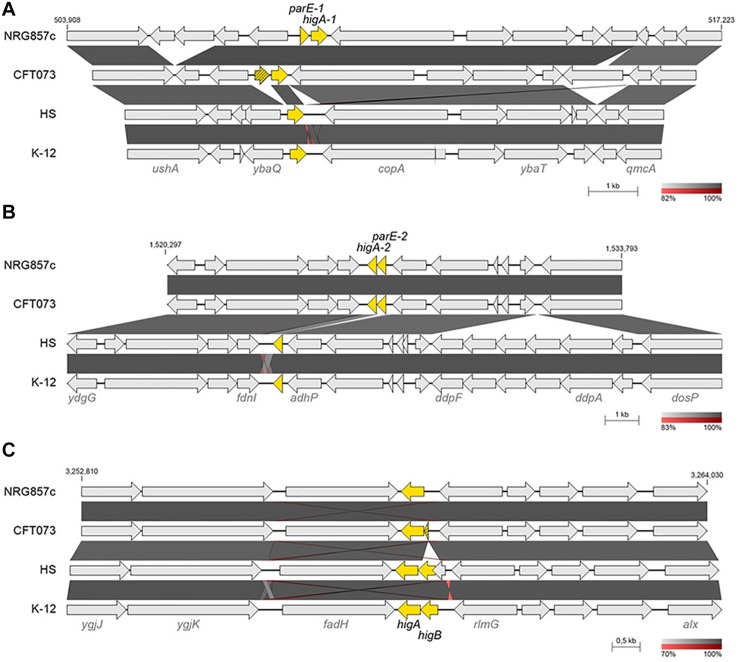
Genomic comparison of *parE*-*higA* loci. Chromosomal regions encoding for *parE*/*higA* loci in NRG857c **(A,B)** and *higBA* locus in MG1655 **(C)** were compared by BLASTN. Highly homologous regions are shaded in gray colors according to the percentage of identity indicated at the legend shown below each figure. TA genes are highlighted in yellow and the coordinates of the DNA segment of NRG857c chromosome used for the comparison are indicated. Compared genomes are same as Figure 1.

Five type II toxin genes (*ccdB-1*, *parE-1*, *parE-2*, *relE-1*, and *parE-3*) are missed at the non-pathogenic *E. coli* chromosomes and shared by AIEC NRG857c and UPEC CFT073 strains (Figures 2B–D, 3).

*ccdAB-1* (TA17) is encoded in the intergenic *folA*-*apaH* region (Figure 2B), a locus known to be plastic and subject to insertion of foreign DNA (Mine et al., 2009). However, in *E. coli* K-12 and the commensal HS strains the intergenic *folA*-*apaH* region covers 77 bp, lacking of *ccdAB* genes (Figure 2B). CcdAB was the first identified plasmidial TA system (CcdAB_F_ from the F plasmid) (Ogura and Hiraga, 1983) and homologs have been found on the chromosome of a large number of bacteria, many of them pathogenic (Pandey and Gerdes, 2005; Saavedra De Bast et al., 2008). CcdB-1 and CcdB_CFT__073_ are identical and share only 34.7% amino acid identity with the plasmidial CcdB_F_. Residue W99 is reported to be crucial for CcdB toxicity in *E. coli* (Loris et al., 1999) and a W99R amino acid substitution is known to compromise *in vitro* the lethal effect of CcdB in *S.* Typhimurium (Lobato-Marquez et al., 2015). CcdB-1 and CcdB_CFT__073_ have an aspartic residue at that position (Supplementary Figure S4) however, if this variation affects the toxicity of CcdB in these pathogenic bacteria is unknown.

On the other hand, *relE-1*/*yiaG-1* system (TA27) are encoded at a more variable locus, but in synteny with CFT073; however, K-12 and HS lack these TA genes along with their immediately bordering genes, though the flanking *yiiD* and *fdhE* genes are conserved (Figure 2C).

Similarly to *ccdAB-1*, *parE-3*/*yiaG-2* (TA30) is encoded in the intergenic *nadR*-*attA* region, but in *E. coli* K-12 and HS strains the intergenic *nadR*-*attA* region covers 307 bp, lacking of these TA genes (Figure 2D).

The examples above highlight the hypothesis that TA systems form genomic islet that could be considered as MGE themselves (Hayes and Van Melderen, 2011), and according to our findings they would be important for pathogenic bacteria as they have been maintained or inserted at these chromosomal positions, opposite to what we found on commensal strains.

Apart of ParE-3 toxin, we identified two additional ParE toxins encoded at the NRG857c chromosome and shared by the UPEC CFT073 (Figure 1B). All of them have an inverted gene order regarding the classic TA operon, with the toxin gene encoded first and, different to ParE-3, the partner antitoxin of ParE-1 and ParE-2 corresponds to a HigA antitoxin (TA19 and TA20) (Figure 3). When we looked in detail the sequences, it was revealed that the *parE-1* homologous in CFT073 corresponds to a pseudogene and only the *higA* antitoxin gene is present at HS and K-12 (Figure 3A). Likewise, only the antitoxin partner of *parE-2* is encoded by HS and K-12 at that locus (Figure 3B). Conversely, *E. coli* K-12 and HS code for a complete HigBA TA system at the intergenic *fadH*-*rlmG* region; but NRG857c and CFT073 encode an orphan *higA* antitoxin gene (Figure 3C), which was not detected by our current method as we focused our search in toxin genes.

### Putative TA Systems Exclusives of NRG857c

*mazF-1* and *hipA-2* toxin genes seem to be unique to NRG857c according to our preliminary comparative analysis (Figure 1B) so we could speculate that these TAs may have an important role in this pathogen.

**FIGURE 4:**
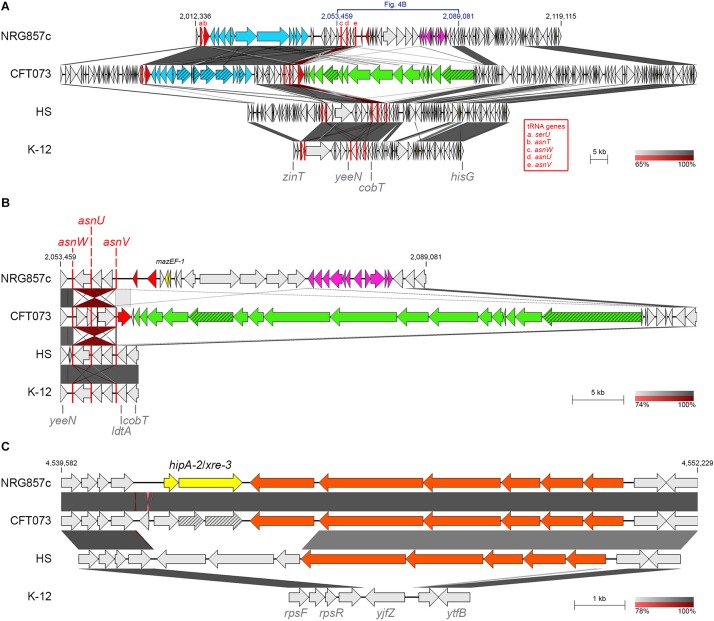
Putative TA systems exclusives of NRG857c. BLASTN genomic comparison of *mazEF-1* **(A,B)** and *hipA-2*/*xre-3* **(C)** loci. **(A)** Comparison of the chromosomal region containing *mazEF-1* in NRG857c; a zoom of the region from *yeeN* to *cobT* is shown at **(B)**. Yersiniabactin gene cluster and the colibactin genomic island are in light blue and green, respectively; PTS genes are in pink; integrase/transposase genes are in red; pseudogenes are in a scratched pattern; tRNA genes are shown as red rectangles (not at scale) and are named with letters a-e according to the red legend. **(C)** Genomic comparison of the region coding *hipA-2*. Fatty acids metabolism genes are in orange. In all figures highly homologous regions are shaded in gray colors according to the percentage of identity indicated at the legend shown below each figure. TA genes are highlighted in yellow and the coordinates of the DNA segment of NRG857c chromosome used for the comparison are indicated. Compared genomes are same as Figure 1.

Two copies of the MazEF system were identified at the NRG857c chromosome. One of them (*mazEF-2*, TA24) resembles the classic *mazEF* locus of *E. coli* encoded at the intergenic *relA*-*mazG* region (Masuda et al., 1993) and MazF-2 shares 99.1% identity with its K-12 homologous (not shown). The second *mazEF* locus in NRG857c (*mazEF-1*, TA22) is exclusive of this AIEC strain and missing from CFT073, HS and K-12 chromosomes. Detailed sequence analysis revealed that *mazEF-1* is encoded inside a region of genome plasticity that include the genomic islands GI-17 to GI-19 described by Nash et al. (2010) (Figures 1A, 4A,B). MazEF was described as the chromosomal homologs of the *pem* locus responsible for the stable maintenance of plasmid R100 (Masuda et al., 1993). MazF-1 shares only 25.7% amino acid identity with the chromosomal MazF_K–__12_ (and with MazF-2), but it shares 76.4% amino acid identity with the plasmidial PemK toxin from R100 plasmid. *mazEF-1* is encoded close to three tRNA genes (*asnW*, *asnU*, and *asnV*, Figure 4B) and a transposase gene, within an approximately 28 kb chromosomal segment, spanning from *asnV* to *ldtA*, exclusive of this strain (Figure 4B). At this locus, CFT073 has a colibactin genomic island known to integrate at the *asnW* gene (Putze et al., 2009). However, NRG857c does not carry the colibactin genomic island and instead it seems to encode a different genomic island coding the *mazEF-1* system, a transposase/recombinase and a phosphoenolpyruvate-dependent sugar phosphotransferase system (PTS) (pink genes in Figures 4A,B). PTS is a major carbohydrate transport system in bacteria that link the uptake and metabolism of sugars, as it catalyzes the phosphorylation of incoming sugar substrates coupling it with translocation across the cell membrane (Postma et al., 1993). However, to our knowledge, the contribution of these genes to AIEC pathogenicity has not been studied.

Two copies of *hipA* are also encoded by NRG857c. In addition to the *hipBA-1* locus described above, NRG857c encodes a second *hipA* toxin gene (*hipA-2*), with a different antitoxin partner (*hipA-2*/*xre-3*, TA28); both are pseudogenes at CFT073 (Figure 4C). *hipA-2* is encoded between ribosomal protein genes (*rps* genes) and a fatty acids metabolism gene cluster shared by CFT073 and partially by HS, but completely absent at K-12 (Figure 4C). However, as we detailed below, opposite to *mazF-1*, *hipA-2* is not exclusive of AIEC and it is shared by a plethora of different *E. coli* strains (see below).

### Screening of NRG857c Putative TA Toxin Genes on Sequenced AIEC Strains

**FIGURE 5:**
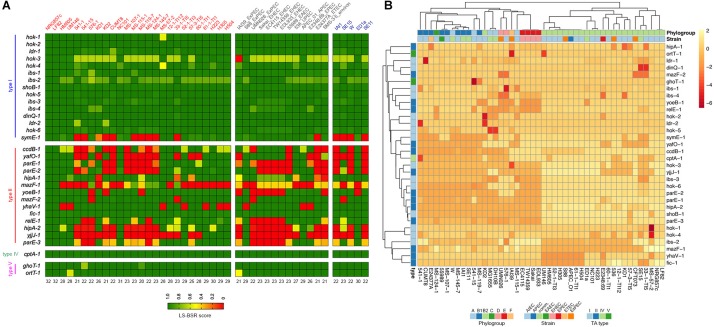
Prevalence of NRG857c putative TA toxins in AIEC and non-AIEC *E. coli* strains. **(A)** Toxin genes were scanned in the genomes by TBLASTN using the LS-BSR pipeline. Achieved LS-BSR score ratios are plotted according to the scale shown at the bottom legend. AIEC strain names are in red, different *E. coli* pathotypes (including an environmental isolate) in gray and commensal strains in blue. Numbers below the image indicate the number of NRG857c toxin genes identified in each strain with a score over 0.7 (from a total of 32, as *hok-2* was considered as one copy). **(B)** Heatmap showing the unsupervised hierarchical clustering of the strains based on their TA repertoire. Columns represent strains and rows toxin gene. Both rows and columns are clustered using correlation distance and average linkage. Annotations on the heatmap are colored according to the bottom legends.

In order to investigate the distribution and conservation of TA toxins in AIEC, we examined the genome sequence of twenty-six diverse characterized AIEC strains available at databases, including four animal isolates (Supplementary Table S1). Strains sequenced by Rakitina et al. (2017) were omitted as their AIEC phenotype was not tested at the time of writing this manuscript. Representative sequenced strains from different *E. coli* pathotypes, as well as environmental and commensal strains, were also included (Figure 5 and Supplementary Table S1).

Surprisingly, except for the LF82 reference strain, we did not found any strain that shares the 33 putative TA toxin genes with NRG857c (Figure 5A). As before, type II toxin genes were the more variable regarding to occurrence and only Fic-1 toxin was coded by all AIEC and non-AIEC strains. The HM605 reference strain was found to lack four TA toxin genes: *ccdB-1*, *mazF-1*, *yhaV-1*, and *hipA-2*. Remarkably and in agreement with our previous findings, *mazF-1* was exclusive of AIEC, but only coded by 8 out of 26 AIEC strains. As the majority of AIEC genomes are drafts, and we did not get deep into their contig nature, we cannot discard *mazF-1* to be plasmidial encoded in some strains.

An additional PCA analyses showed that, based on their TA toxin repertoire, *E. coli* strains cluster according to phylogroups instead of pathotypes (Figure 5B and Supplementary Figure S5). Most B2 strains cluster together, with HM605 apart of LF82 and NRG857c, but within a mostly homogeneous group including ExPEC and avian pathogenic *E. coli* (APEC) strains. Noteworthy, the four EHEC strains (E phylogroup) cluster together into a homogeneous group that shares the same repertoire of TA toxin genes analyzed here.

### AIEC Toxin Genes Respond *in vitro* to Bile Salts and Acid Stress

In order to determine whether AIEC TA toxin genes are transcriptionally active genes able to respond to stress conditions found by the bacteria in the intestine and into the macrophage, toxin mRNA levels were evaluated by qRT-PCR with total RNA extracted from bacteria grown on bile salts and acid conditions (Figure 5). Twelve toxin genes were analyzed, including nine type II and the types IV and V toxin genes. Type I toxin genes were not included as some are present in more than one copy and they share high sequence identity (particularly *hok*, *ldr*, and *ibs* genes), being unable to be amplified by specific oligonucleotides. Control genes (*eutE*, *fimH*, *htrA*, and *dsbA*) known to be upregulated by AIEC under these stress conditions were also included. Furthermore, we included two phylogenetically distant AIEC reference strains in the analysis, NRG857c and HM605, in order to check for variability in their transcriptional responses.

**FIGURE 6:**
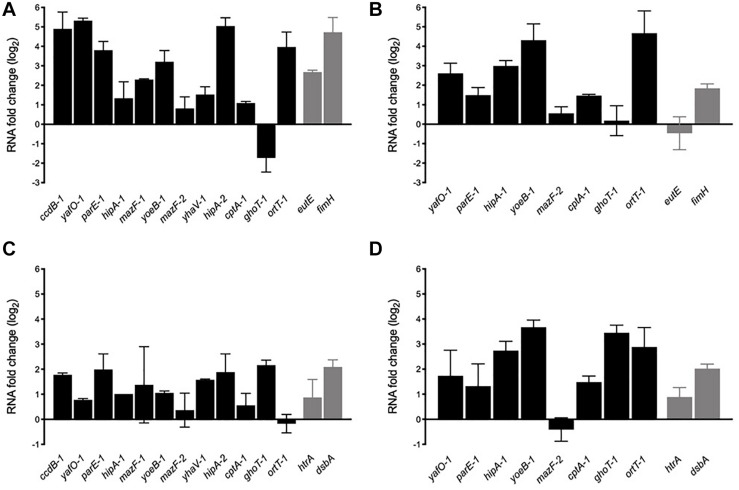
*In vitro* mRNA expression levels of toxin genes in response to bile salts **(A,B)** and acidic conditions **(C,D)**. NRG857c **(A,C)** or HM605 **(B,D)** were grown until early exponential phase and then sub-cultured into fresh LB medium containing 2% bile salts or LB at pH 4.5, and incubated with agitation at 37°C for 2 h. Black bars represent expression levels of toxin genes and gray bars expression levels of control genes. RNA expression levels were normalized using *gapA* as reference gene and are expressed as log_2_ of RNA fold change against the level of expression in LB medium at early exponential phase according to the efficiency-calibrated ΔCt model.

After 2-h growing in the presence of bile salts almost all toxin genes were upregulated (except *ghoT-1*) indicating that they are transcriptionally active and responsive to stress. *ccdB-1*, *yafO-1*, *parE-1*, *yoeB-1*, *hipA-2*, and *ort-1* were highly expressed in NRG857c (3-5 log_2_ fold changes, equivalent to 8-32 fold changes) (Figure 6A). As expected *eutE* and *fimH*, known genes to be upregulated by AIEC LF82 in the presence of bile salts (Chassaing et al., 2013; Delmas et al., 2019), were also upregulated in this strain. A similar toxin expression pattern (except for *ghoT-1*) was observed with HM605 (Figure 6B). *ccdB-1*, *mazF-1*, *yhaV-1*, and *hipA-2* gene expression was not measured in this strain as they are not encoded by HM605 (Figure 5A). However, contrary to NRG857c (Figure 6A) and LF82 (Delmas et al., 2019), expression levels of the control *eutE* gene did not change in HM605 in the presence of bile salts. *ghoT-1*, coding for the type V membrane-damaging toxin GhoT (Wang et al., 2012), was the unique gene down-regulated in NRG857c (does not change its expression levels in HM605). Surprisingly, *ortT-1*, encoding for an orphan GhoT-like toxin (Islam et al., 2015), was one with the highest expression levels (4-5 log_2_ fold changes) in both AIEC strains.

Upregulation found in the presence of bile salts was also observed in acid stress, but with a lower levels (Figures 6C,D). However, contrary to the bile salts condition (Figures 6A,B), under acid stress *ghot-1* was upregulated in both strains. Now, differences were seen with *ortT-1*, which was upregulated only in HM605 (Figure 6D). As expected, control genes *htrA* and *dsbA* whose transcription is known to be upregulated when the bacteria grown under stress conditions similar to those encountered within the phagocytic vacuole, such as acidic medium (Bringer et al., 2005, 2007), were upregulated at pH 4.5 in both strains (Figures 6C,D).

Thus, our results show that TA toxin genes are transcriptionally active and responsive to stress conditions that AIEC could face inside its host, suggesting that TA toxin expression could be important for AIEC to face stress conditions and be able to reach or survive into their target cells. Furthermore, our findings recall that AIEC are diverse bacteria that do not necessary share regulatory mechanisms or stress responses.

### AIEC Upregulates Their Putative TA Toxin Genes Inside the Macrophage

**FIGURE 7:**
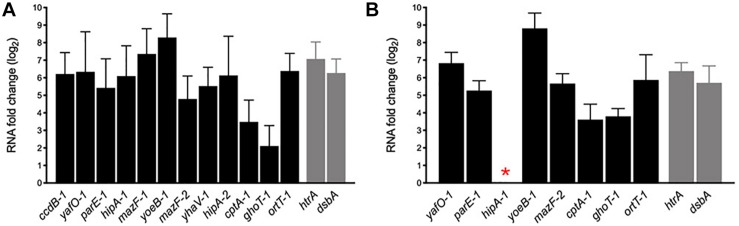
mRNA expression levels of putative toxin genes inside the macrophage. Macrophages J774.A1 were infected with NRG857c **(A)** or HM605 **(B)** and 1 h post infection cells were recovered for RNA extraction. Black bars represent expression levels of toxin genes and gray bars expression levels of controls genes. The red asterisk indicates a gene whose expression could not be detected in HM605. RNA expression levels were normalized using *gapA* as reference gene and are expressed as log_2_ of RNA fold change against the level of expression in LB medium at early exponential phase according to the efficiency-calibrated ΔCt model.

During host infection pathogens are exposed to various stressful conditions, being the intracellular environment of macrophages one of the most survival-threatening. However, in addition to acid stress, such as that we tested *in vitro* (Figures 6C,D), pathogens must face a battery of macrophage antimicrobial responses that include oxidative stress, toxic metal cations, and antimicrobial peptides (Kaufmann and Dorhoi, 2016). AIEC is characterized for its remarkable capacity to survive and replicate inside the macrophage and, in spite of some bacterial genes are known to be upregulated and important in these intracellular conditions, the mechanisms involved are unknown. In order to investigate whether the transcription of the AIEC putative TA toxin genes was regulated by the stress conditions found inside the macrophage, murine J774.A1 macrophages were infected with NRG857c and HM605 strains, RNA was purified after 1 h post infection (see section “Materials and Methods”) and mRNA levels measured by qRT-PCR (Figure 7). As expected, control genes *htrA* and *dsbA* were highly upregulated in both strains. Our results revealed high expression levels of all tested TA toxin genes, even the ort*T-1* gene that did not change its expression in NRG857c at acid pH (Figure 6C). Surprisingly, *hipA-1* expression could not be detected in intramacrophagic HM605, indicating that the transcription of *hipA-1* is severely repressed by conditions found inside the macrophage, although it is highly expressed at pH 4.5 (Figure 6D).

These results indicate that TA toxin genes are transcriptionally regulated inside the macrophage and, in the case that this upregulation leads to toxin activation, they could play key roles in the ability of AIEC to resist macrophage killing, and thus be able to survive and replicate as characteristic of this *E. coli* pathotype.

## Discussion

Most bacteria harbor multiple TA systems on their chromosome but their abundance could vary even between closely related organisms with few to no systems being universally conserved in a given species (Fraikin et al., 2020). It was postulated that pathogenic bacteria possess more TA systems than non-pathogenic-related species (Pandey and Gerdes, 2005). Nowadays that assumption is not well supported, as some clinical isolates such as ExPEC (Norton and Mulvey, 2012) and, as we revealed here, AIEC NRG857c, have a reduced number of TA systems compared to the non-pathogenic *E. coli* strain MG1655. Though, pathogens such as *M. tuberculosis*, encode a significantly expanded repertoire of TA systems (Slayden et al., 2018). Nevertheless, the emergent developing of new strategies is continuously identifying new promising TA candidates, so those TA repertoires undoubtedly will increase. Recently, a new resource for the discovery of TA systems in known bacterial genomes, TASmania (Akarsu et al., 2019), has been released. However, as our analysis was done before this new database become available, we compared our results with the TASmania output for NRG857c. With the exception of the orphan type V toxin OrtT-1 and the not annotated genes (Supplementary Material and Supplementary Figures S6–S8), all the TA systems identified in this work were also detected by TASmania, and no additional putative TA systems were identified at the NRG857c chromosome.

In agreement with and in addition to previous finding, our results show that AIEC are a diverse group of bacteria that not necessary share transcriptional responses. They do not have a common TA toxin repertoire and their TA toxin genes are not evenly regulated in different environments or AIEC strains. Significant differences in the prevalence of type II TA toxin genes among AIEC and other *E. coli* pathotypes were noted, so we conclude that the TA repertoire identified here is exclusive of NRG857c and LF82, rather than a feature of the AIEC pathotype. Nevertheless, our PCA analyses showed that, based on their TA toxin repertoire, strains cluster according to phylogroups, mainly B2 strains (Figure 5B and Supplementary Figure S5). Indeed, a potential link between chromosomal type II TA systems and *E. coli* phylogeny was previously suggested (Fiedoruk et al., 2015). Our clustering analysis, comprising a complete TA toxin repertoire and not only type II toxins, confirm that linkage.

Noteworthy, *mazF-1* seems to be a toxin gene exclusive to AIEC, though we must to compare a higher number of genomes to conclude this. Remarkable MazF-1 is closer in sequence to the plasmidial PemK toxin than to the chromosomal MazF version. In addition, *mazEF-1* is part of a genomic island inserted at a tRNA-Asn gene, a known hot spot for insertion of MGEs such as the colibactin genomic island in CFT073 (Figures 4A,B). At this locus NRG857c codes a different genomic island coding a PTS system involved in the uptake and metabolism of sugars (Postma et al., 1993). *hipA-*2 is also encoded within a region of genome plasticity coding functions related to fatty acids metabolism (Figure 4C), absent at the non-pathogenic K-12 strain. Indeed, a recent work that tracked the adaptive evolution of AIEC in a murine model revealed that consumption of short-chain fatty acid confer fitness advantages to AIEC in the gut (Elhenawy et al., 2019). So, as it is known that TA systems could participate in virulence contributing to the maintenance of virulence factors-encoding MGE ([Bibr B64][Bibr B19]; Lobato-Marquez et al., 2016b), the role of these genomic islands as well as their TA systems in AIEC pathogenesis, are aspects that must be achieved.

TA systems are important for niche-specific colonization and stress resistance of UPEC, and it has been demonstrated that individual TA systems can have profound effects on bacterial fitness within distinct host environments (Norton and Mulvey, 2012). Here we have shown *in vitro* evidence of changes on TA toxin genes expression under different stress conditions. For example acid pH and intramacrophage conditions boosted the expression of *ghoT-1* in NRG857c, but this gene is downregulated with bile salts; opposite to the transcriptional response of *ortT-1*, coding for an orphan GhoT-like toxin, which was upregulated under bile salts conditions as well as within the macrophage, but does not change its expression at acid pH (at least in NRG857c) (Figures 6, 7). Another TA toxin, MqsR, regulates GhoT via post-transcriptional differential mRNA cleavage, resulting in a regulatory hierarchy where one TA system controls another (Wang et al., 2013). We were unable to find homologs of the MqsRA system in NRG857c so different regulatory mechanisms must to control *ghoT* expression in this strain. However, as we did not measure antitoxin gene expression levels and considering the transcriptional autoregulation of TA systems by conditional cooperativity (particularly for type II) (Harms et al., 2018), the biological significance of these transcriptional changes needs further experimental evidence.

On the other hand, the role of TA systems might not only be dependent on the environmental conditions but also on their genetic context. We found genomic TA systems forming genomic islets, such as *ccdAB-1* and *parE-3*/*yiaG-2* (Figures 2B,C), as well as differences at the genomic context of some TA systems were revealed. For instance, *hipBA-1* encodes close to the F9 fimbrial operon (Figure 2A). So far F9 operon has been reported to be important for the colonization of calves by *E. coli* O157:H7 and O26 (Dziva et al., 2004; Van Diemen et al., 2005), to mediate adherence to bovine epithelial cells (Low et al., 2006) and to have a role in biofilm formation in CFT073 (Ulett et al., 2007). Immediately upstream genes could have a role in the transcriptional regulation of fimbrial operons, and we speculate that *hipBA-1* should have an important role at that specific chromosomal position, as it has been preserve at this locus independently of the F9 operon integrity.

Our findings reveal that in addition to the TA repertoire, the genetic context where these genes are encoded would discriminate between pathogenic and non-pathogenic strains, in addition to affect their activities and/or roles.

As Crohn’s disease is a chronic illness, and one of the most remarkable characteristics of AIEC is its capacity to persist within target cells, the contribution of TA systems to AIEC persistence and/or stress management must undoubtedly be explore. For example it has been speculated that the plasmidial CcdB in *Salmonella* could play a different role than the classic plasmid maintenance and instead, it could be connected to the activation of persistent phenotypes (Di Cesare et al., 2016). The same could be the case for the CcdB variants found in AIEC strains and other pathogenic bacteria, such as the UPEC CFT073. On the other hand, HipA is a serine-protein kinase (Schumacher et al., 2009) that phosphorylates glutamyl-tRNA synthase, inhibiting protein synthesis and contributing to persister formation (Germain et al., 2013; Kaspy et al., 2013); described high-persister *hipA* mutants cause multidrug tolerance (Moyed and Bertrand, 1983; Schumacher et al., 2015). It is known that *hipA* mutations, including *hipA7*, are important players in clinically relevant *E. coli* multi drug tolerance infections. HipA-1 lacks the described mutations associated to the high-persister phenotype (not shown); however, HipA-1 is 98.2% identical with its K-12 counterpart and curiously some of its amino acid variations are also share by HipA_CFT__073_ (not shown).

As our analysis was only based on sequence analysis, and taking into account that upregulation of toxin genes not necessarily means activity of the toxin (as the antitoxin could still being active and inhibiting toxin activity), it is important to do functional experiments to complement our findings. In addition, the molecular characterization of toxin variants that were identified here, such as HipA-1 and CcdB-1, will be relevant to identify their real contribution to persistence and/or stress management in AIEC.

Thus, although the biological role of these putative TA systems at the pathogenicity of AIEC remains to be determined, altogether our results have shown that TA systems could be attractive bacterial factors to explore and to get a better comprehension of the pathogenesis of AIEC and its contribution to the chronicity of Crohn’s disease.

## Data Availability Statement

The datasets generated for this study are available on request to the corresponding author.

## Author Contributions

PB planned the experiments, performed the experiments, analyzed the data, and designed and prepared the manuscript. RV planned the experiments and supervised the work. PB and RV were involved in the final editing of the manuscript.

## Conflict of Interest

The authors declare that the research was conducted in the absence of any commercial or financial relationships that could be construed as a potential conflict of interest.

## Footnotes

1 https://db-mml.sjtu.edu.cn/TAfinder/index.php

2 snapgene.com
