# Supplementary material for: Repertoire and Diversity of Toxin – Antitoxin Systems of Crohn’s Disease-Associated Adherent-Invasive Escherichia coli. New Insight of T his Emergent E. coli Pathotype
Source: Front Microbiol. 2020 May 6;11:807. doi: 10.3389/fmicb.2020.00807 (PMC7232551; doi:10.3389/fmicb.2020.00807)
Supplement: Supplementary file 1 [file Data_Sheet_1.PDF]

Supplementary Material

Curation of not annotated/incorrectly annotated toxin genes

Hok genes According to protein alignment, it was clear that two genes (NRG857c_02625 and NRG857c_07840) were incorrectly annotated as they encode for Hok proteins with N-terminal extension absent in the references Hok proteins (Fig S6A Hok-2.1 and Hok 4). To our knowledge, there are not Hok proteins that contain these extra N-terminal amino acids, so we concluded that they were incorrectly annotated. The correct genes must to cover from nucleotide 577,301..577,453 and c1,626,517..1,626,672, for hok-2.1 and hok-4, respectively. On the other hand, the gene corresponding to hok-3 was missing from the annotation available at NCBI and it covers the nucleotides c1,466,912..1,467,052.

Ldr genes By multiple amino acid sequence alignment we notice that ldr-1 was annotated giving a protein with 9 extra amino acids absent in the references Ldr proteins (Fig. S7). Thus, we conclude that the correct ldr-1 must cover from nucleotide 1,288,750 to 1,288,857.

shoB-1 The shoB-1 identified at NRG857c was missing from the annotation and must to cover from nucleotide 2,685,649 to 2,685,729.

dinQ We found that the dinQ locus in E. coli K-12 is conserved in NRG857c, but the corresponding gene was missing from the annotation. We denoted this gen dinQ-1 and found that must be encoded between the nucleotides 3,663,342 to 3,663,425 and coding a protein 96.3 % identical to its K-12 homologous.

ibs/sib genes E. coli K-12 encoded five copies of the ibs/sib system19, but they were missed from the annotation in NRG857c. ibsA/sibA and ibsB/sibB systems are encoding in tandem in the yegL-mdtA intergenic region at E. coli K-12. At the same locus in NRG857c the ibs/sib genes were omitted from the annotation. The region from 2,175,707 to 2,175,763 contain an ORF (ibs-1) coding for a protein that share 55.6% and 94.4% identity with IbsA and IbsB, respectively; the homologous of ibsB is encoded between the nucleotides 2,176,038 to 2,176,091s (ibs-2) and codes for a protein that share 66.67% and 88.9% identity with IbsA and IbsB, respectively (Fig. S8). The ibsC/sibC system is coded in the fau (former ygfA)-serA intergenic region at K-12; the corresponding homologue in NRG857c is coded between nucleotides 3,031,097 and 3,031,156 (ibs-3). ibsD/sibD and ibsE/sibE systems are encoding in tandem in the ygiK-rfaE intergenic region at E. coli K-12. At the same locus in NRG857c the ibs/sib genes were omitted from the annotation. The region from 3,220,142 to 3,220,201 contains an ORF (ibs-4) coding for a protein that share 94.7% identity with IbsE. The fifth copy, ibsD is missing in NRG857c and we speculate that could be a sequencing/assembly error.
