# Supplementary material for: Repertoire and Diversity of Toxin – Antitoxin Systems of Crohn’s Disease-Associated Adherent-Invasive Escherichia coli. New Insight of T his Emergent E. coli Pathotype
Source: Front Microbiol. 2020 May 6;11:807. doi: 10.3389/fmicb.2020.00807 (PMC7232551; doi:10.3389/fmicb.2020.00807)
Supplement: Supplementary file 2 [file Data_Sheet_2.PDF]

Table S1. Sequenced AIEC and non-AIEC strains used in the comparative analysis.

Strain	Host	Accession number	Pathotype
LF82	Human	NC_011993.1	AIEC
NRG857c	Human	NC_017634.1	AIEC
HM605	Human	AJWU00000000	AIEC
UM146	Human	NC_017632	AIEC
541_1	Human	AJWR00000000	AIEC
541_15	Human	AJWQ00000000	AIEC
576_1	Human	AJWS00000000	AIEC
KD1	Dog	AJWO00000000	AIEC
KD2	Dog	AJWP00000000	AIEC
CUMT8	Mouse	AJWV00000000	AIEC
NC101	Mouse	AEFA00000000	AIEC
MS-107-1	Human	40713.ADWV01000001-ADWV01000072.nuc	AIEC
MS-115-1	Human	47227.ADTL01000001-ADTL01000423.nuc	AIEC
MS-119-7	Human	40709.ADWU01000001-ADWU01000130.nuc	AIEC
MS-124-1	Human	40707.ADWT01000001-ADWT01000176.nuc	AIEC
MS-145-7	Human	40703.ADWS01000001-ADWS01000143.nuc	AIEC
MS-57-2	Human	47209.ADUG01000001-ADUG01000408.nuc	AIEC
12-1-TI12	Human	10.4225/13/56F08C5B5F0FE	AIEC
33-1-TI5	Human	10.4225/13/56F08C5B5F0FE	AIEC
52-1-TI3	Human	10.4225/13/56F08C5B5F0FE	AIEC
57-3-TI5	Human	10.4225/13/56F08C5B5F0FE	AIEC
60-1-TI1	Human	10.4225/13/56F08C5B5F0FE	AIEC
61-1-TI1	Human	10.4225/13/56F08C5B5F0FE	AIEC
H223	Human	10.4225/13/56F08C5B5F0FE	AIEC
H305	Human	10.4225/13/56F08C5B5F0FE	AIEC
H504	Human	10.4225/13/56F08C5B5F0FE	AIEC
IAI39	Human	NC_011750.1	ExPEC (UPEC)
S88	Human	CU928161.2	ExPEC (UPEC)
UMN026	Human	NC_011751.1	ExPEC (UPEC)
CFT073	Human	NC_004431.1	ExPEC (UPEC)
APEC_O1	Avian	NC_008563.1	ExPEC (APEC)
536	Human	NC_008253.1	ExPEC (UPEC)
Sakai	Human	NC_002695.1	EHEC
EC4115	Human	NC_011353.1	EHEC
TW14359	Human	NC_013008.1	EHEC
EDL933	Human	NZ_CP008957.1	EHEC
55989	Human	NC_011748.1	EAEC
E2348:69	Human	NC_011601.1	EPEC
E24377A	Human	NC_009801.1	ETEC

SMS-3-5	Human	NC_010498.1	environmental
IAI1	Human	NC_011741.1	commensal
SE15	Human	NC_013654.1	commensal
W	Human	NC_017635.1	commensal
ED1a	Human	NC_011745.1	commensal
SE11	Human	NC_011415.1	commensal
HS	Human	NC_009800.1	commensal
DH10B	Human	NC_010473.1	commensal
MG1655	Human	NC_000913.3	commensal
