# Supplementary material for: Repertoire and Diversity of Toxin – Antitoxin Systems of Crohn’s Disease-Associated Adherent-Invasive Escherichia coli. New Insight of T his Emergent E. coli Pathotype
Source: Front Microbiol. 2020 May 6;11:807. doi: 10.3389/fmicb.2020.00807 (PMC7232551; doi:10.3389/fmicb.2020.00807)
Supplement: Supplementary file 3 [file Data_Sheet_3.PDF]

Table S2. Oligonucleotides used at this work.

Name	Sequence	Use
qCcdB1_F	GCCGCGTTTCCCTTTGTTA	forward primer to amplify *ccdB-1*
qCcdB1_R	GGGCGAATACGGCTAAATCG	reverse primer to amplify *ccdB-1*
qYafO1_F	ATCCATTTCCGCCACAGTTG	forward primer to amplify *yafO-1*
qYafO1_R	ATGGCAATGAGCAACCATGC	reverse primer to amplify *yafO-1*
qParE1_F	ACACAAGGGATTGCGTGACT	forward primer to amplify *parE-1*
qParE1_R	GCTGCATCAATCACAGCGAG	reverse primer to amplify *parE-1*
qHipA1_F	TTTGCTGCTGGCGAAAGAAC	forward primer to amplify *hipA-1*
qHipA1_R	CTCCTGTGGCAAGCGAAGTA	reverse primer to amplify *hipA-1*
qMazF1_F	TACCCGTTGTTGTTCCCGTT	forward primer to amplify *mazF-1*
qMazF1_R	CGGGCTCCCATATCAATGGT	reverse primer to amplify *mazF-1*
qYoeB1_F	GGCAGGAAGCAGATAAGCGA	forward primer to amplify *yoeB-1*
qYoeB1_R	CGGGACCAGAAACCTGACAA	reverse primer to amplify *yoeB-1*
qMazF2_F	CTGTTGTCCTGAGTCCGTTCA	forward primer to amplify *mazF-2*
qMazF2_R	TCAGCTAACGCTACGCCATC	reverse primer to amplify *mazF-2*
qYhaV1_F	TTGCCGAAGTCGAGGCATTA	forward primer to amplify *yhaV-1*
qYhaV1_R	TGACCGTGATATGCTCCTCA	reverse primer to amplify *yhaV-1*
qHipA2_F	GGGTTGGGGCTCTTCGATAC	forward primer to amplify *hipA-1*
qHipA2_R	CTAGCCCTTTCCAGACTCGC	reverse primer to amplify *hipA-1*
qCptA1_F	CACCCCGTTATGGATGGTGT	forward primer to amplify *cptA-1*
qCptA1_R	TTGACGATGCTCCACTCCTG	reverse primer to amplify *cptA-1*
qGhoT1_F	TGGTGTGAACATATCCTTTGTCA	forward primer to amplify *ghoT-1*
qGhoT1_R	ATGCCACAGGCAGACTCATT	reverse primer to amplify *ghoT-1*
qOrtT1_F	CGCCCGTACATGTCTCTCTA	forward primer to amplify *ortT-1*
qOrtT1_R	TGTTGCCCCCACCAGAAAG	reverse primer to amplify *ortT-1*
qEutE_F	CTCAACCAGGCGATTGTTGC	forward primer to amplify *eutE*
qEutE_R	GCCGATACCCGGAAACTTGA	reverse primer to amplify *eutE*
qFimH_F	GGTCGGTAAATGCCTGGTCA	forward primer to amplify *fimH*
qFimH_R	GGTTTTGCCCCACATTCACG	reverse primer to amplify *fimH*
qHtrA_F	TATCAACACCGCGATCCTCG	forward primer to amplify *htrA*
qHtrA_R	ACGTTTCACCTGGCCGTATT	reverse primer to amplify *htrA*
qDsbA_F	CGGAAGGCGTGAAGATGACT	forward primer to amplify *dsbA*
qDsbA_R	GCCTTCAAACAGCGGAACTG	reverse primer to amplify *dsbA*
qGapA_F	GCTCGTAAACACATCACCGC	forward primer to amplify *gapA*
qGapA_R	AGCGTTGGAAACGATGTCCT	reverse primer to amplify *gapA*
