# Supplementary material for: Repertoire and Diversity of Toxin – Antitoxin Systems of Crohn’s Disease-Associated Adherent-Invasive Escherichia coli. New Insight of T his Emergent E. coli Pathotype
Source: Front Microbiol. 2020 May 6;11:807. doi: 10.3389/fmicb.2020.00807 (PMC7232551; doi:10.3389/fmicb.2020.00807)
Supplement: Supplementary file 4 [file Data_Sheet_4.PDF]

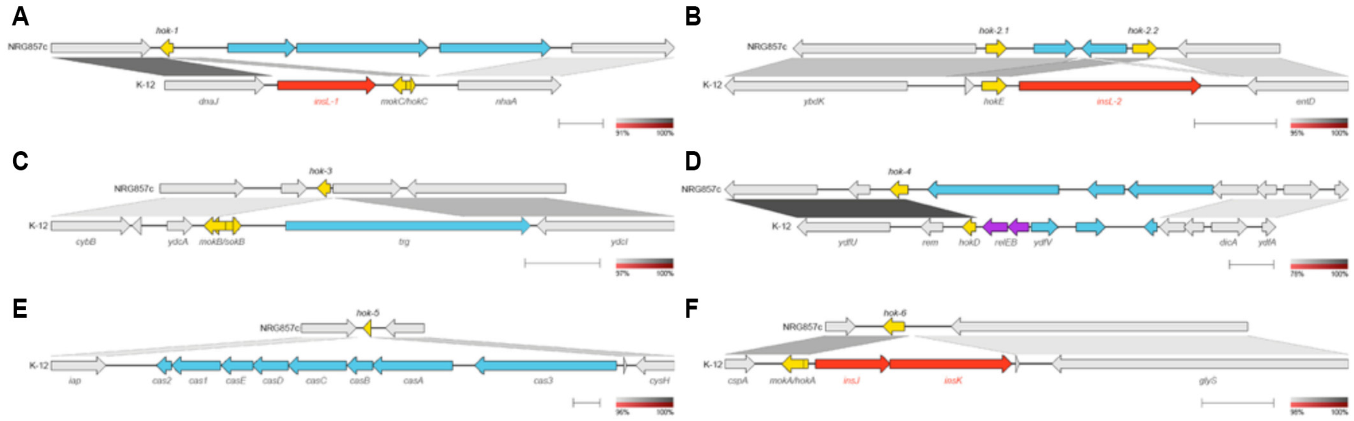

Figure S1. *hok* genes encoded by NRG857c. **(A)** *hok-1* **(B)** *hok-2* **(C)** *hok-3* **(D)** *hok-4* **(E)** *hok-5* **(F)** *hok-6*. *hok* genes (or *hok-sok* systems in K-12) are highlighted in yellow. Transposase genes are in red. *relBE* system is in purple Non-shared genes are in light blue. Genomes of NRG857c (GenBank NC_017634.1) and K-12 MG1655 (GenBank NC_000913) were compared by BLASTN and homologous regions are shaded in grey colors according to the percentage of identity indicated at the legend. The scale bar represents 0.5 kb.
