# Supplementary material for: Repertoire and Diversity of Toxin – Antitoxin Systems of Crohn’s Disease-Associated Adherent-Invasive Escherichia coli. New Insight of T his Emergent E. coli Pathotype
Source: Front Microbiol. 2020 May 6;11:807. doi: 10.3389/fmicb.2020.00807 (PMC7232551; doi:10.3389/fmicb.2020.00807)
Supplement: Supplementary file 5 [file Data_Sheet_5.PDF]

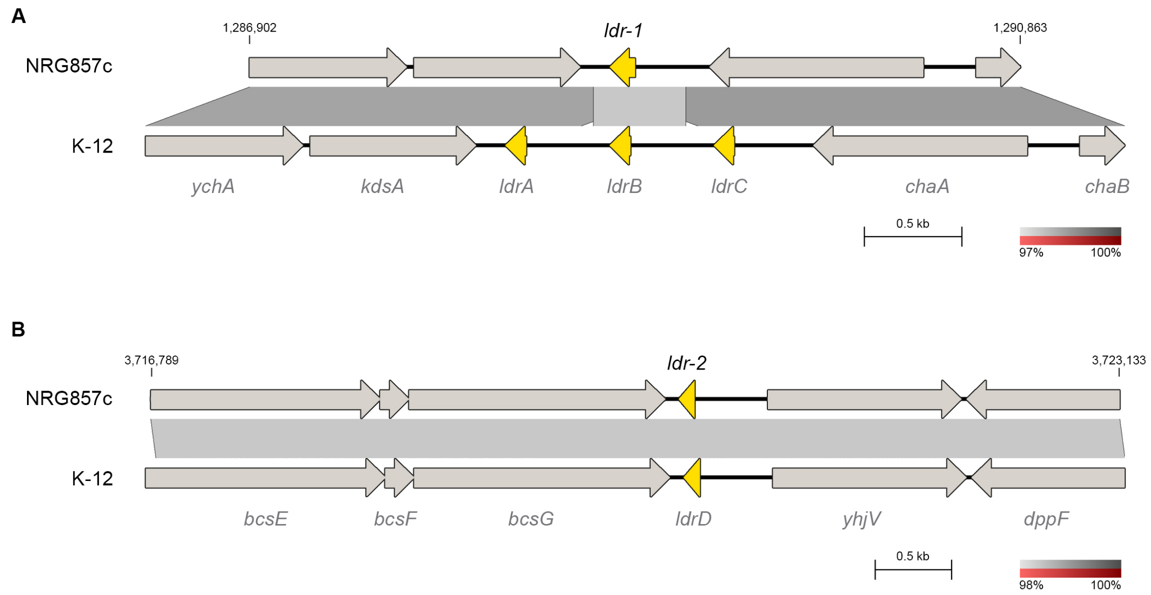

Figure S2. Genomic comparison of chromosomal regions encoding for *ldr* genes. *ldr-1* **(A)** and *ldr-2* **(B)** loci were compared by BLASTN and highly homologous regions are shaded in grey colors according to the percentage of identity indicated at the legend shown below each figure. *ldr* genes are highlighted in yellow and the coordinates of the DNA segment of NRG857c chromosome used for the comparison are indicated. Genomes are same as in Fig. S1.
