# Supplementary material for: Repertoire and Diversity of Toxin – Antitoxin Systems of Crohn’s Disease-Associated Adherent-Invasive Escherichia coli. New Insight of T his Emergent E. coli Pathotype
Source: Front Microbiol. 2020 May 6;11:807. doi: 10.3389/fmicb.2020.00807 (PMC7232551; doi:10.3389/fmicb.2020.00807)
Supplement: Supplementary file 6 [file Data_Sheet_6.PDF]

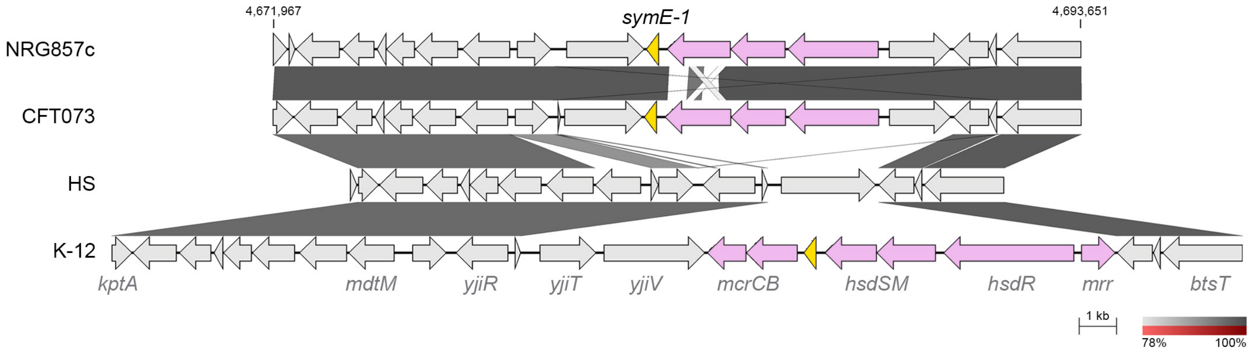

Figure S3. Genomic comparison of the *symE-1* locus. Genomes (same as Fig. 1) were compared by BLASTN and highly homologous regions are shaded in grey colors according to the percentage of identity indicated at the legend shown below the figure. The *symE* toxin genes are highlighted in yellow and restriction-modification genes in pink. The coordinates of the DNA segment of NRG857c chromosome used for the comparison are indicated.
