# Supplementary material for: Repertoire and Diversity of Toxin – Antitoxin Systems of Crohn’s Disease-Associated Adherent-Invasive Escherichia coli. New Insight of T his Emergent E. coli Pathotype
Source: Front Microbiol. 2020 May 6;11:807. doi: 10.3389/fmicb.2020.00807 (PMC7232551; doi:10.3389/fmicb.2020.00807)
Supplement: Supplementary file 7 [file Data_Sheet_7.PDF]

```
CcdB_F          MQFKVYTYK-RESRYRLFVDVQSDIIDTPGRRMVIPLASARLLSDKV-SRELYPVV-HIG    57
CcdB_pSLT       MQFKVYTCK-RESRYRLFVDVQSDIIDTPGRRMAVPLVSARLLSEKV-PRDLYPVM-HIG    57
CcdB_CFT073     MQFTVYRSRGRNAAFPFVIDVTSDIIGEINRRIVIPLTPIERFSRIRPPERLNPILLLVD    60
CcdB-1          MQFTVYRSRGRNAAFPFVIDVTSDIIGEINRRIVIPLTPIERFSRIRPPERLNPILLLVD    60
                ***.**  : *:: : :.:** ****.  .**:.:**.  . :*     . * *::  :.

CcdB_F          DESWRMMTTQMVSVPVSVIGEEVADLSHRENDIKNAINLMFWGI    101
CcdB_pSLT       DEPYRLLTTDMTSVPATVIGEEVADLSLRENDIKNAINLMFRGI    101
CcdB_CFT073     GKEYVLMTHETATVPVNALGTKFCDASAHRTLIKGALDFMLDGI    104
CcdB-1          GKEYVLMTHETATVPVNALGTKFCDASAHRTLIKGALDFMLDGI    104
                .: : ::* : .:**...:* :..* * :.. **.*:::*: **
```

Figure S4. Multiple amino acid sequence alignment of CcdB proteins. CcdB proteins encoded by F plasmid (GenBank AAA96062), pSLP plasmid (WP_001159863), UPEC CFT073 (WP_001160966) and NRG857c (YP_006118417) were alignment by CLUSTAL O(1.2.4). Identical amino acid residues are highlighted in black. Residue W99, reported to be crucial for CcdB toxicity in *E. coli*, along with its variation, are highlighted in yellow.
