# Supplementary material for: Repertoire and Diversity of Toxin – Antitoxin Systems of Crohn’s Disease-Associated Adherent-Invasive Escherichia coli. New Insight of T his Emergent E. coli Pathotype
Source: Front Microbiol. 2020 May 6;11:807. doi: 10.3389/fmicb.2020.00807 (PMC7232551; doi:10.3389/fmicb.2020.00807)
Supplement: Supplementary file 8 [file Data_Sheet_8.PDF]

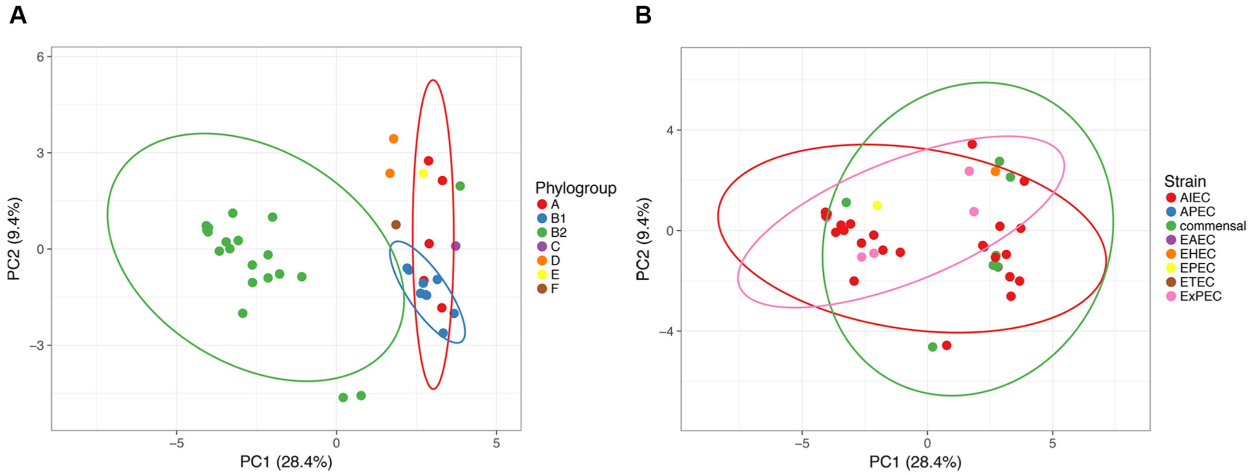

Figure S5. Scatter plot of the first two principal components of a PCA analysis based on the TA toxin genes in AIEC and non-AIEC *E. coli* strains. Data were analysed according to phylogroup (A) or type of strain (pathotypes) (B). Prediction were done by ClustVis and ellipses are such that with a probability 0.95. Percentages of the total variances described by the first two extracted factors are given on the axes.
