# Supplementary material for: Repertoire and Diversity of Toxin – Antitoxin Systems of Crohn’s Disease-Associated Adherent-Invasive Escherichia coli. New Insight of T his Emergent E. coli Pathotype
Source: Front Microbiol. 2020 May 6;11:807. doi: 10.3389/fmicb.2020.00807 (PMC7232551; doi:10.3389/fmicb.2020.00807)
Supplement: Supplementary file 9 [file Data_Sheet_9.PDF]

A

```
Hok-6_YP_006121884.1        ---------------------------------MPQKYRLLSLIVICFTLLFFTWMIRDS      27
HokA                        ---------------------------------MPQKYRLLSLIVICFTLLFFTWMIRDS      27
Hok-5_YP_006121047.1        ---------------------------------MLTKYALVAIIVLCCTVLGFTLMVGDS      27
Hok-2.1_YP_006118889.1      MKPQEIFLSTLGLPLQTRTIWMVVPSSHGGNINMLTKYALVAVIVLCLTVLGFTLLVGDS      60
Hok-2.2_YP_006118891.1      ---------------------------------MLTKYALVAVIVLCLTVLGFTLLVGDS      27
HokE                        ---------------------------------MLTKYALAAVIVLCLTVLGFTLLVGDS      27
Hok-1_YP_006118382.1        --------------------------------MKQHKAMIVALIVICITAVVAALVTRKD      28
HokC                        --------------------------------MKQHKAMIVALIVICITAVVAALVTRKD      28
Hok-4_YP_006119926.1        -------------MLDTCRLASYVPKGKEKQAMKQQKAMLIALIVICLTVIVTALVTRKD      47
HokD                        --------------------------------MKQQKAMLIALIVICLTVIVTALVTRKD      28
Hok_R1                      -------------------------------MKLPRSSLVWCVLIVCLTLLIFTYLTRKS      29
Hok-3                       -------------------------------LATTANPR-PC---ESNTILTF-TLTRQS      24
HokB                        ---------------------------------MKHNPLVVCLLIICITILTFTLLTRQT      27
                                                                    .    .    . * :    :  .

Hok-6_YP_006121884.1        LCELHIKQESYELAAFLACKLKE 50
HokA                        LCELHIKQESYELAAFLACKLKE 50
Hok-5_YP_006121047.1        LCELSIRERGMEFKAVLAYESKK 50
Hok-2.1_YP_006118889.1      LCEFTVKERNIEFKAVLAYEPKK 83
Hok-2.2_YP_006118891.1      LCEFTVKERNIEFKAVLAYEPKK 50
HokE                        LCEFTVKERNIEFKAVLAYEPKK 50
Hok-1_YP_006118382.1        LCEVHIRTGQTEVAVFTAYESE- 50
HokC                        LCEVHIRTGQTEVAVFTAYESE- 50
Hok-4_YP_006119926.1        LCEVRIRTGQTEVAVFTAYEPEE 70
HokD                        LCEVRIRTGQTEVAVFTAYEPEE 51
Hok_R1                      LCEIRYRDGHREVAAFMAYESGK 52
Hok-3                       LYELRFRDGDKEVAALMACTSRE 47
HokB                        LYELRFRDGDKEVAALMACTSR- 49
                            * *.  :    *. .. *
```

B

1: Hok-6_YP_006121884.1	100.00	100.00	48.00	44.00	44.00	44.00	34.69	34.69	38.00	38.00	34.00	33.33	34.69
2: HokA	100.00	100.00	48.00	44.00	44.00	44.00	34.69	34.69	38.00	38.00	34.00	33.33	34.69
3: Hok-5_YP_006121047.1	48.00	48.00	100.00	80.00	80.00	78.00	36.73	36.73	36.00	36.00	34.00	26.67	32.65
4: Hok-2.1_YP_006118889.1	44.00	44.00	80.00	100.00	100.00	98.00	30.00	30.00	31.43	35.29	34.62	21.28	28.57
5: Hok-2.2_YP_006118891.1	44.00	44.00	80.00	100.00	100.00	98.00	30.61	30.61	36.00	36.00	36.00	22.22	28.57
6: HokE	44.00	44.00	78.00	98.00	98.00	100.00	28.57	28.57	36.00	36.00	36.00	22.22	26.53
7: Hok-1_YP_006118382.1	34.69	34.69	36.73	30.00	30.61	28.57	100.00	100.00	82.00	82.00	36.00	26.67	38.78
8: HokC	34.69	34.69	36.73	30.00	30.61	28.57	100.00	100.00	82.00	82.00	36.00	26.67	38.78
9: Hok-4_YP_006119926.1	38.00	38.00	36.00	31.43	36.00	36.00	82.00	82.00	100.00	100.00	36.54	27.66	32.65
10: HokD	38.00	38.00	36.00	35.29	36.00	36.00	82.00	82.00	100.00	100.00	37.25	28.26	32.65
11: Hok_R1	34.00	34.00	34.00	34.62	36.00	36.00	36.00	36.00	36.54	37.25	100.00	44.68	53.06
12: Hok-3	33.33	33.33	26.67	21.28	22.22	22.22	26.67	26.67	27.66	28.26	44.68	100.00	77.27
13: HokB	34.69	34.69	32.65	28.57	28.57	26.53	38.78	38.78	32.65	32.65	53.06	77.27	100.00

Figure S6. Multiple amino acid sequence alignment of Hok proteins. **(A)** Hok proteins identified in AIEC NRG857c (Hok-1 to 6) were aligned along with reference Hok proteins. For Hok-3, the translated sequenced obtained from the nucleotides c1,466,912..1,467,052 of NRG857c was used. The Hok proteins from *E. coli* K-12 MG1655 (HokA-E; GenBank YP_026229, YP_025301, YP_025292, NP_416080 and YP_025295, respectively) and the plasmidial Hok protein from the *E. coli* plasmid R1 (GenBank CAA29259) were included in the alignment. Sequences considered as not part of Hok proteins are highlighted in yellow. Alignment was done by CLUSTAL O(1.2.4). **(B)** Amino acid percent identity matrix generated by Clustal2.1. In grey the percent identity between themself and in yellow the highest percent identity found for Hok proteins from NRG857c.
