# Supplementary material for: Repertoire and Diversity of Toxin – Antitoxin Systems of Crohn’s Disease-Associated Adherent-Invasive Escherichia coli. New Insight of T his Emergent E. coli Pathotype
Source: Front Microbiol. 2020 May 6;11:807. doi: 10.3389/fmicb.2020.00807 (PMC7232551; doi:10.3389/fmicb.2020.00807)
Supplement: Supplementary file 10 [file Data_Sheet_10.PDF]

A

```
LdrB        ---------MTLAQFAMIFWHDLAAPIILAGIITAAIVGWWRNRK      35
LdrA        ---------MTLAQFAMIFWHDLAAPIILAGIITAAIVSWWRNRK      35
LdrC        ---------MTLAQFAMIFWHDLAAPIILAGIITAAIVSWWRNRK      35
Ldr-1       MLSLQQGGYMTLAQFAMIFWHDLAAPIILAGIITAAIVGWWRNRK      44
LdrD        ---------MTFAELGMAFWHDLAAPVIAGILASMIVNWLNKRK      35
Ldr-2       ---------MTLAELGMAFWHDLAAPVIAGILASMIVNWLNKRK      35
                     **:*::.* ********::***::: **.* .:**
```

B

1: LdrB	100.00	94.29	94.29	97.14	57.14	60.00
2: LdrA	94.29	100.00	100.00	97.14	57.14	60.00
3: LdrC	94.29	100.00	100.00	97.14	57.14	60.00
4: Ldr-1	97.14	97.14	97.14	100.00	57.14	60.00
5: LdrD	57.14	57.14	57.14	57.14	100.00	97.14
6: Ldr-2	60.00	60.00	60.00	60.00	97.14	100.00

Figure S7. Multiple amino acid sequence alignment of Ldr proteins. **(A)** Ldr proteins identified in AIEC NRG857c (Ldr-1 and Ldr-2) were aligned along with reference Ldr proteins from *E. coli* K-12: LdrA (GenBank YP_025297.1), LdrB (YP_025298), LdrC (YP_025299), LdrD (YP_026227), Ldr-1 (YP_006119607) and Ldr-2 (YP_006121859). The sequence considered as not part of Ldr proteins in NRG857c is highlighted in yellow. Alignment was done by CLUSTAL O(1.2.4). **(B)** Amino acid percent identity matrix generated by Clustal2.1. In grey the percent identity between themself and in yellow the highest percent identity found for Ldr proteins from NRG857c.
