# Supplementary material for: Repertoire and Diversity of Toxin – Antitoxin Systems of Crohn’s Disease-Associated Adherent-Invasive Escherichia coli. New Insight of T his Emergent E. coli Pathotype
Source: Front Microbiol. 2020 May 6;11:807. doi: 10.3389/fmicb.2020.00807 (PMC7232551; doi:10.3389/fmicb.2020.00807)
Supplement: Supplementary file 11 [file Data_Sheet_11.PDF]

A

```
Ibs-2                   MMK-WIIIVVLLVISFPAY 18
IbsB_YP_002791248.1     MMK-LLIIVVLLVISFPAY 18
Ibs-1                   MMK-LLIIVVLLVISYPAY 18
IbsA_YP_002791247.1     MMKHVIILVILLVISFQAY 19
IbsE_YP_002791257.1     MMKLVIILVVLLLLSFPTY 19
Ibs-4                   MMKFVIILVVLLLLSFPTY 19
IbsC_YP_002791255.1     MMRLVIILIVLLLISFSAY 19
Ibs-3                   MMRLVIILIVLLLISFPAY 19
IbsD_YP_002791256.1     MMKLVIILIVLLLVSFAAY 19
                        **:  :*:::**::*: :*
```

B

1: Ibs-2	100.00	88.89	83.33	77.78	72.22	72.22	66.67	72.22	66.67
2: IbsB_YP_002791248.1	88.89	100.00	94.44	72.22	66.67	66.67	61.11	66.67	61.11
3: Ibs-1	83.33	94.44	100.00	66.67	61.11	61.11	55.56	61.11	55.56
4: IbsA_YP_002791247.1	77.78	72.22	66.67	100.00	68.42	68.42	68.42	68.42	68.42
5: IbsE_YP_002791257.1	72.22	66.67	61.11	68.42	100.00	94.74	73.68	78.95	78.95
6: Ibs-4	72.22	66.67	61.11	68.42	94.74	100.00	68.42	73.68	73.68
7: IbsC_YP_002791255.1	66.67	61.11	55.56	68.42	73.68	68.42	100.00	94.74	84.21
8: Ibs-3	72.22	66.67	61.11	68.42	78.95	73.68	94.74	100.00	84.21
9: IbsD_YP_002791256.1	66.67	61.11	55.56	68.42	78.95	73.68	84.21	84.21	100.00

Figure S8. Multiple amino acid sequence alignment of Ibs proteins. **(A)** Ibs proteins from *E. coli* K-12 and NRG857c were alignment by CLUSTAL O(1.2.4). **(B)** Amino acid percent identity matrix generated by Clustal2.1.
